# Low-frequency noise induced by cation exchange fluctuation on the wall of silicon nitride nanopore

**DOI:** 10.1038/s41598-020-65530-y

**Published:** 2020-05-26

**Authors:** Kazuma Matsui, Yusuke Goto, Itaru Yanagi, Rena Akahori, Michiru Fujioka, Takeshi Ishida, Takahide Yokoi, Tatsuo Nakagawa, Ken-ichi Takeda

**Affiliations:** 1 Center for Technology Innovation – Healthcare, Research and Development Group, Hitachi, Ltd., 1-280 Higashi-Koigakubo, Kokubunji, Tokyo, 185-8601 Japan; 2 Department of Biotechnology and Life Science, Tokyo University of Agriculture and Technology, Koganei, Tokyo, 184-8588 Japan; 3 Bio Systems Design Department, Hitachi High-Tech Corporation, 882 Ichige, Hitachinaka, Ibaraki, 312-8504 Japan

**Keywords:** Biosensors, Nanopores

## Abstract

Nanopore-based biosensors have attracted attention as highly sensitive microscopes for detecting single molecules in aqueous solutions. However, the ionic current noise through a nanopore degrades the measurement accuracy. In this study, the magnitude of the low-frequency noise in the ionic current through a silicon nitride nanopore was found to change depending on the metal ion species in the aqueous solution. The order of the low-frequency noise magnitudes of the alkali metal ionic current was consistent with the order of the adsorption affinities of the metal ions for the silanol surface of the nanopore (Li <Na <K < Rb <Cs). For the more adsorptive alkaline earth metal ions (Mg and Ca), the low-frequency noise magnitudes were as low as those for Li ions. This tendency, *i.e*., metal ions having a very high or low adsorption affinity causing a reduction in low-frequency noise, suggests that the low-frequency noise was induced by the exchange reactions between protons and metal ions occurring on the silanol surface. In addition, the low-frequency noise in the ionic current remained low even after replacing the CaCl_2_ aqueous solution with a CsCl aqueous solution, indicating that Ca ions continued being adsorbed onto silanol groups even after removing the aqueous solution.

## Introduction

With the recent scaling down of electronic devices, the noise caused by charge fluctuations is becoming apparent and greatly affects the reliability of electronic devices such as field-effect transistors. In these devices, trapping and detrapping of an electron or hole at a localized defect state can cause a measurable current change^1,2^, which increases the magnitude of the current noise in the low-frequency region from approximately 1 Hz to 100 Hz.

Similar noise has also been reported in the study of the ionic current through a nanopore^3–14^. A nanopore device is used to characterize the geometrical and electrical features of a molecule (such as DNA) passing through the nanopore by measuring the ionic current change through the nanopore, but low-frequency noise can inhibit accurate measurements in biosensing applications. In contrast to the semiconductor device mentioned above, however, charge carriers are neither electrons nor holes but ions in an aqueous solution. Therefore, the cause of the observed low-frequency noise can be interpreted as an ionic current fluctuation.

On the basis of their constituent materials, nanopore technologies are classified into two types: biological^15–18^ and solid-state nanopores^19^. Biological nanopores are formed by pore-forming proteins such as Mycobacterium smegmatis porin A and α-hemolysin. The latest explanation for the low-frequency noise in the current through biological nanopores is related to conformational flexibilities of the nanopore structural constituents^5,6^. By modifying the hydrophobic-hydrophilic ratio of the block copolymer membrane, the conformational changes caused by membrane-protein interactions are successfully suppressed, leading to a reduction in low-frequency noise^7^. On the other hand, solid-state nanopores are formed by inorganic materials. For example, semiconductor-related materials such as silicon nitride (SiN_x_)^19–27^ and graphene^28,29^ are often used. As for graphene nanopores, mechanical fluctuation of the membrane is proposed as an underlying cause of the low-frequency noise^28,29^. As for SiN_x_ nanopores, the low-frequency noise of ionic current is thought to be induced by surface charge fluctuation on the nanopore wall^10,14^. Wen, C. *et al*. investigated the dependence of the low-frequency noise on the pore diameter by using various SiN_x_ pores with different diameters in the range of 7 to 200 nm^14^. They concluded that the dominant cause for the low-frequency noise was due to the surface charge fluctuation when the diameter of the pore was small (especially, less than 20 nm). The amount of the surface charges and the amount of the conduction ions in the nanopore became comparable as the diameter of the pore became smaller. As a result, the ionic current through the nanopore could largely fluctuate in response to the surface charge fluctuation. In this study, we investigated on very small nanopores with a diameter of 1-2 nm, and the dominant low-frequency noise component was assumed to be the surface charge fluctuation on the nanopore wall. An already advocated explanation for the surface charge fluctuation in a SiN_x_-based nanopore is the transition model between the trapping and detrapping of a single proton (H^+^) on the surface of the nanopore wall^10,14^. The fluctuation in the ionic current through a nanopore originates from the following proton exchange reaction of the silanol (SiOH) groups^10^:

1 $${{\rm{SiO}}}^{-}{+{\rm{H}}}^{+}\rightleftarrows {\rm{SiO}}\mbox{--}{\rm{H}}\,$$

2 $${{\rm{SiO}}\mbox{--}{\rm{H}}+{\rm{H}}}^{+}\rightleftarrows {{\rm{SiOH}}}_{2}^{+}$$

This model predicts the pH dependence of the magnitude of the low-frequency noise^10^. In this paper, we report an unexplored phenomenon whereby the low-frequency noise depends on not only the protons but also on the metal ions (M^+^) in the solution, and this phenomenon cannot be explained by the proton fluctuation model.

## Results and discussion

### Difference in low-frequency noise depending on cationic species

According to previously reported fabrication procedures^22–27^, we prepared a SiN_x_ membrane with a thickness of 6 nm and sandwiched the membrane between two reservoirs, including 1 mol/L MCl_x_ aqueous solutions (M = Li, Na, K, Rb, Cs, Mg, and Ca). By applying a voltage to the membrane with two Ag/AgCl electrodes, a nanopore was fabricated by utilizing dielectric breakdown^20,22^. The measured conductances of MCl_x_ aqueous solutions were Li: 74.2; Na: 87.3; K: 112.0; Rb: 115.2; Cs: 114.6; Mg: 108.5; and Ca:132.2 mS/cm, respectively. The size of the nanopore was adjusted so that the ionic current through the nanopore was almost 1 nA at 0.2 V regardless of the type of the aqueous solution used. This is because the low-frequency noise depends on the magnitude of the ionic current^8,29^. The diameters of the fabricated nanopores were in the range of 1 to 2 nm.

**Figure 1:**
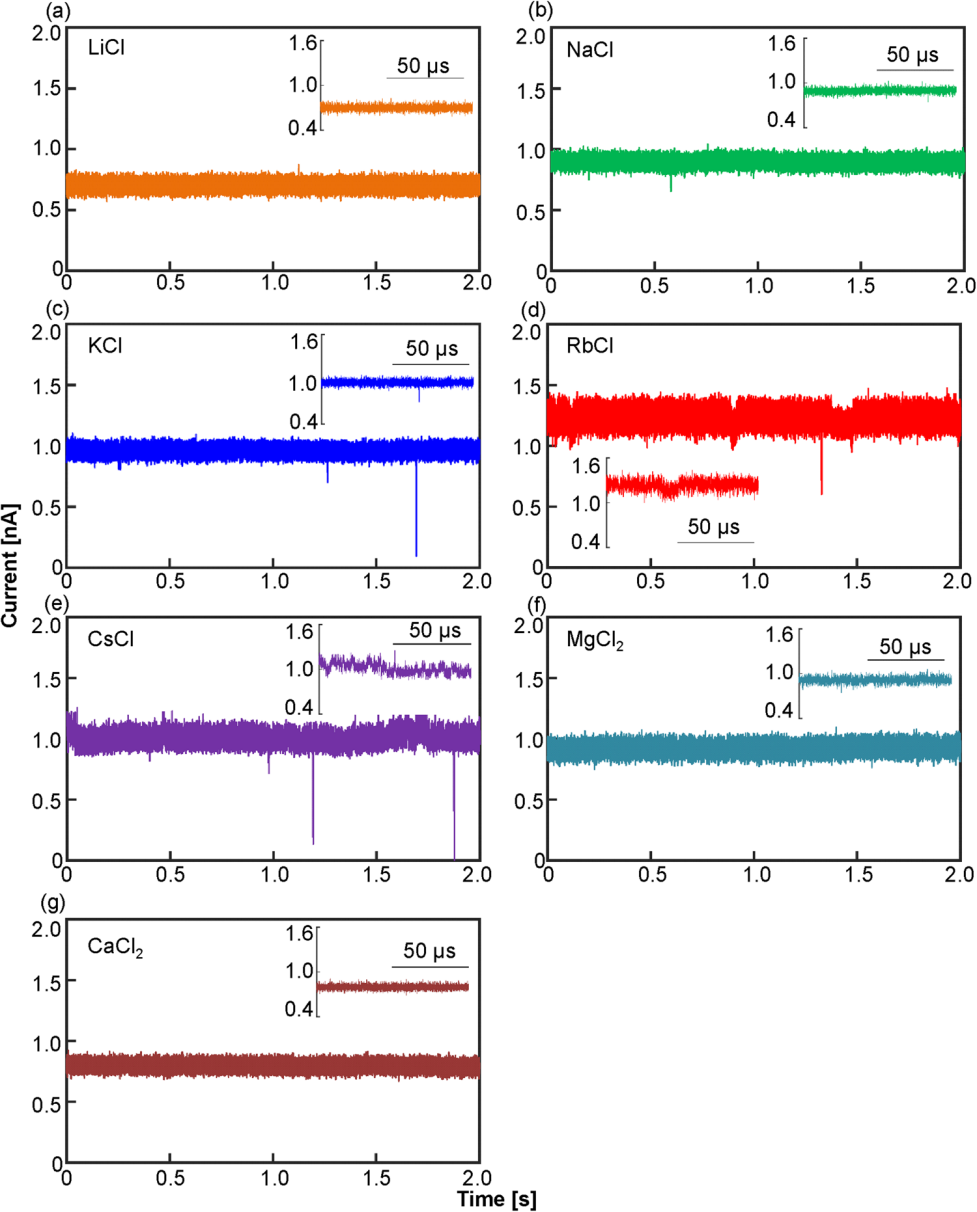
Time traces of the ionic currents flowing through the nanopore in LiCl, NaCl, KCl, RbCl, CsCl, MgCl_2_, and CaCl_2_ aqueous solutions. All data were acquired at 0.2 V.

**Figure 2:**
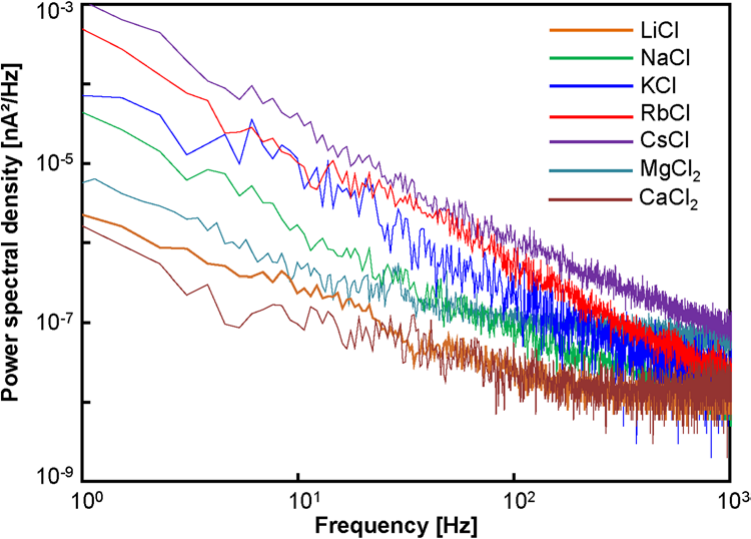
PSDs of the ionic currents flowing through the nanopore in LiCl, NaCl, KCl, RbCl, CsCl, MgCl_2_, and CaCl_2_ aqueous solutions. All data were acquired at 0.2 V.

Figures 1 and 2 show typical time traces and power spectral densities (PSDs) of the currents using the MCl_x_ aqueous solutions at 0.2 V. Apparently, the currents fluctuated unstably with certain alkali metal ions (such as K, Rb, and Cs). On the other hand, the currents were relatively stable with other alkali metal ions (such as Li and Na). The currents with alkaline earth metal ions (Mg and Ca) remained similarly stable. The PSD of each ionic current (Fig. 2) also reveals that the low-frequency noise below a frequency of 1 kHz is relatively low with Li, Na, Mg, and Ca.

The dependence of the low-frequency noise on the metal ion species has not been previously reported, and the conventional model based on protonization^4,10,14^ cannot sufficiently explain this dependency.

**Figure 3:**
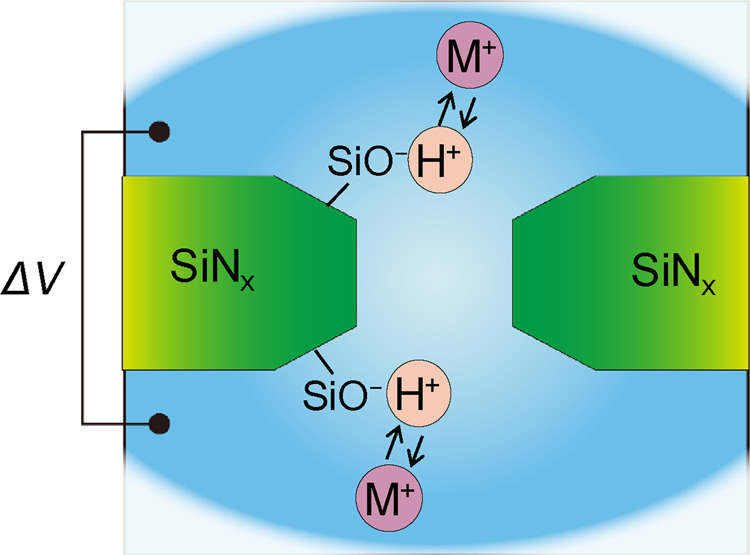
Schematic image of the low-frequency noise model due to the exchange reactions between metal ions and protons on the silanol surface of the SiN_x_ nanopore.

To interpret this phenomenon, we hypothesized that the following exchange reaction between the protons and metal ions on the silanol surface^30–32^ likely generates ionic current fluctuations (Fig. 3):

3 $${{\rm{SiO}}\mbox{--}{\rm{H}}+{\rm{M}}}^{+}\rightleftarrows {{\rm{SiO}}\mbox{--}{\rm{M}}+{\rm{H}}}^{+}$$

Figure 3 shows the exchange reactions at multiple reaction sites. The low-frequency noise in current was attributed to the superposition of the current fluctuations caused by the exchange reactions at multiple reaction sites. Each current fluctuation derived from each reaction site was thought to be a quantized fluctuation like a telegraph noise with a stochastic duration. In addition, the magnitude of each fluctuation could vary depending on the position of the reaction site in the nanopore. Therefore, the superposition of such the fluctuations with various amplitudes and dwell times could generate even large and slow current fluctuations. Some large pulse-like current fluctuations (*i.e*., impulse noises) were observed in KCl, RbCl, and CsCl ionic currents (in Fig. 1). At the present, we cannot provide a definitive explanation for the cause of the large impulse noises. The simultaneous adsorption of multiple metal ions to contiguous reaction sites can be considered as a possible cause. Although the magnitude of the current fluctuation due to the adsorption of each metal ion is small, the simultaneous adsorption can cause a large fluctuation. Another possible cause is related to the position of the exchange reaction site. When the exchange reaction occurs in the vicinity of the narrowest part in the nanopore, the large current fluctuation can be induced.

The ease of the exchange reaction at each reaction site depends on the difference between the binding energies of SiO-H and SiO-M. When the binding energy between SiO^-^ and H^+^ is much larger than the binding energy between SiO^-^ and M^+^, protons stably continue being adsorbed onto the silanol groups. In this situation, exchange reactions between protons and metal ions rarely occur, leading to a reduction in the low-frequency noise. As the binding energies of SiO-H and SiO-M become closed to each other, the exchange reaction can occur frequently, leading to an increase in the low-frequency noise. Furthermore, when the binding energy of SiO-M is much larger than that of SiO-H, the exchange reaction is also inactivated, and the low-frequency noise is reduced.

**Figure 4:**
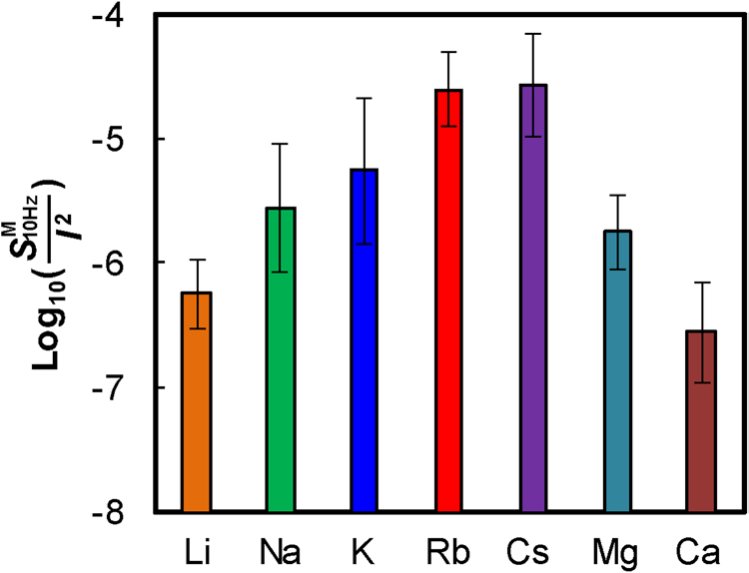
Low-frequency noise magnitudes (${{S}}_{{\rm{10Hz}}}^{{\rm{M}}}$/*I*^2^) of the LiCl, NaCl, KCl, RbCl, CsCl, MgCl_2_, and CaCl_2_ ionic currents through the nanopores. Each data point represents the average ± standard deviation of the logarithmic low-frequency noise magnitude measured in five different nanopores.

To quantitatively evaluate the magnitude of the low-frequency noise, we examined the magnitude of the PSD at 10 Hz (*S*_10Hz_ [nA^2^/Hz]) normalized by the squared value of the ionic current (*I* [nA]). Figure 4 shows the low-frequency noise magnitude (${{S}}_{{\rm{10Hz}}}^{{\rm{M}}}$/*I*^2^) for several MCl_x_ aqueous solutions measured at 0.2 V. Each data point is the average value of the measurements from five different nanopores. We can confirm the following tendency: for alkali metal ions, ${{S}}_{{\rm{10Hz}}}^{{\rm{M}}}$/*I*^2^ increases with increasing atomic number (from Li to Cs). This tendency is consistent with the one whereby the adsorption affinity of the metal ions for silanol groups increases with increasing atomic number (from Li to Cs)^30–32^, indicating that the exchange reaction is activated with increasing adsorption affinity. For alkaline earth metal ions, the low-frequency noise magnitudes were relatively low compared to ${{S}}_{{\rm{10Hz}}}^{{\rm{Rb}}}$/*I*^2^ and ${{S}}_{{\rm{10Hz}}}^{{\rm{Cs}}}$/*I*^2^. This result can be interpreted by the following explanation: in contrast to alkali metal ions, which have one positive net charge, alkaline earth metal ions have two positive net charges. Therefore, alkaline earth metal ions have a higher adsorption affinity for silanol groups. In this case, two following exchange reactions were thought to occur:

4 $${{\rm{2SiO}}\mbox{--}{\rm{H}}+{\rm{M}}}^{2+}\rightleftarrows {{\rm{SiO}}\mbox{--}{\rm{M}}\mbox{--}{\rm{OSi}}+{\rm{2H}}}^{+}$$

5 $${{\rm{SiO}}\mbox{--}{\rm{H}}+{\rm{M}}}^{2+}\rightleftarrows {{\rm{SiO}}\mbox{--}{\rm{M}}}^{+}{+{\rm{H}}}^{+}$$

In the above reactions, the bindings of SiO-M^+^ and SiO-M-OSi were thought to be more stable than the binding of SiO-H. Therefore, once alkaline earth metal ions adsorb onto the silanol groups, the ions hardly desorb from them thereafter. Consequently, the exchange reaction between protons and alkaline earth metal ions infrequently occurs, resulting in a reduction in the low-frequency noise.

As presented in SI-A, we measured the low frequency noises when the two (*cis* and *trans*) chambers were filled with different aqueous solutions. Fig. S1(b) shows power spectral densities of the ionic current when one side of the chambers was filled with LiCl aqueous solution while the other side was filled with CsCl aqueous solution. The magnitude of the low- frequency noise differed depending on the polarity of the voltage. In the voltage condition where Cs ionic current flowed through the nanopore, the low frequency noise was larger than in the case where Li ionic current flowed through the nanopore. This result also supports our hypothesis wherein the magnitude of low-frequency noise depends on the cationic species in the nanopore.

**Figure 5:**
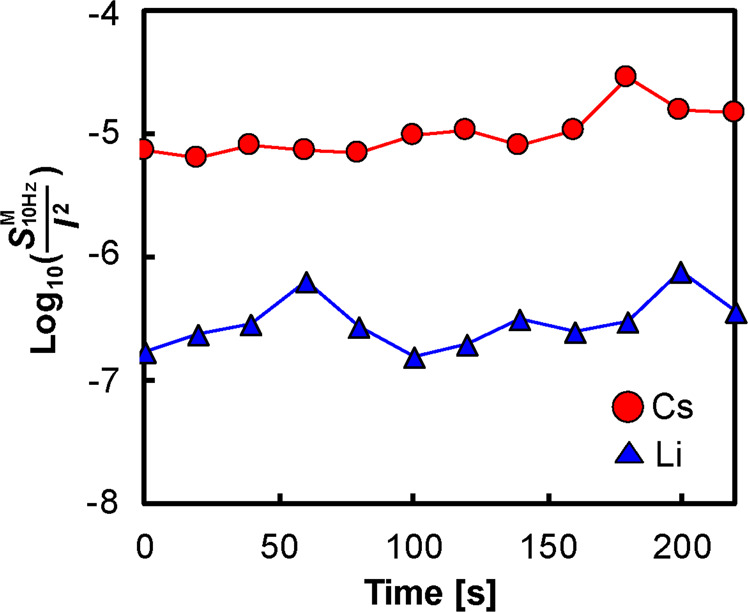
Long-term dependency of the low-frequency noise magnitude (${{S}}_{{\rm{10Hz}}}^{{\rm{M}}}$/*I*^2^) of the LiCl and CsCl ionic current through the nanopores.

We examined whether the low-frequency noise magnitude changed notably over time. Figs. S2 and S3 show the PSDs and time traces over 240 s obtained with CsCl and LiCl aqueous solutions, respectively. While the LiCl ionic current remained stable at any point, the CsCl ionic current largely fluctuated throughout the measurement. Figure 5 shows the time dependency of the low-frequency noise magnitudes (${{S}}_{{\rm{10Hz}}}^{{\rm{Cs}}}$/*I*^2^ and ${{S}}_{{\rm{10Hz}}}^{{\rm{Li}}}$/*I*^2^) monitored every 20 s. Each low-frequency noise magnitude did not change substantially over time, and ${{S}}_{{\rm{10Hz}}}^{{\rm{Cs}}}$/*I*^2^ was higher than ${{S}}_{{\rm{10Hz}}}^{{\rm{Li}}}$/*I*^2^ at any point. The standard deviations (SDs) of the logarithmic low-frequency noise magnitudes in Fig. 5 are 0.20 for Li and 0.18 for Cs, which are much smaller than the SDs obtained from the results in Fig. 4, i.e., 0.28 for Li and 0.42 for Cs. This result indicates that the variation in low-frequency noise over time is smaller than the chip-to-chip variation.

### Low-frequency noise reduction

**Figure 6:**
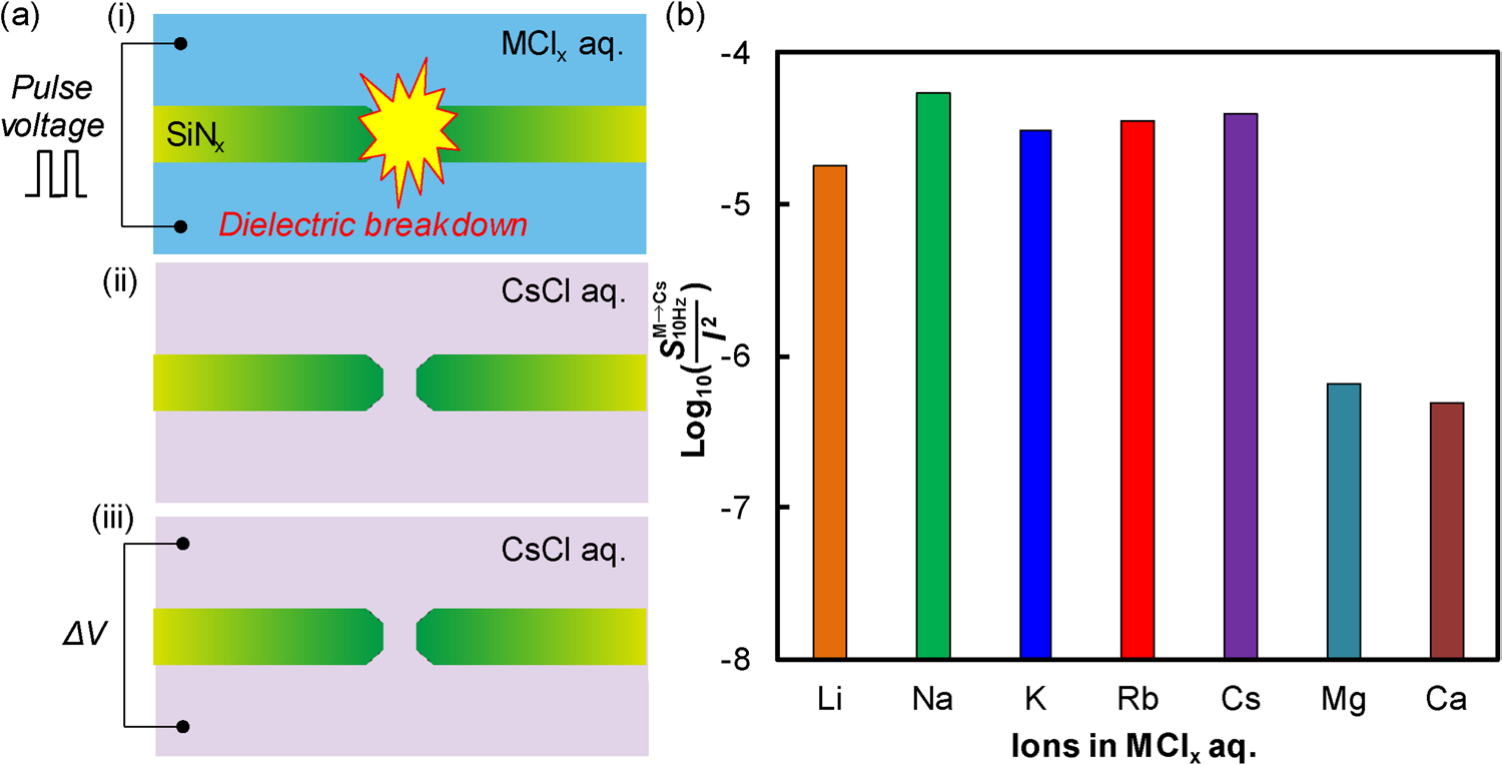
(**a**) Schematic images of the low-frequency noise measurement procedure including the following three steps: (i) nanopore fabrication by utilizing dielectric breakdown in a MCl_x_ aqueous solution, (ii) replacement with a CsCl aqueous solution, and (iii) measurement of the CsCl ionic current through the nanopore at 0.3 V. (**b**) Low-frequency noise magnitudes (${S}{}_{{\rm{10Hz}}}^{{\rm{M}}\to {\rm{Cs}}}$/*I*^2^) of the CsCl ionic currents through the nanopores fabricated by utilizing dielectric breakdown in MCl_x_ aqueous solutions.

We also examined whether alkaline earth metal ions continued being adsorbed onto the silanol groups even after removing the aqueous solution. To this end, the following procedure was executed: (i) a nanopore was fabricated by utilizing dielectric breakdown in a 1 mol/L MCl_x_ aqueous solution (M = Li, Na, K, Rb, Cs, Mg, and Ca) (Fig. 6(a-i)), (ii) the MCl_x_ aqueous solution was replaced with a 1 mol/L CsCl aqueous solution (Fig. 6(a-ii)), and (iii) the ionic current flowing through the nanopore was measured at 0.3 V (Fig. 6(a-iii)). Figure 6(b) shows the low-frequency noise magnitude (${{S}}_{{\rm{10Hz}}}^{{\rm{M}}\to {\rm{Cs}}}$/*I*^2^) of the CsCl ionic current through the nanopore after replacing the aqueous solution. The horizontal axis indicates the MCl_x_ aqueous solution used for nanopore fabrication. The dependence of ${{S}}_{{\rm{10Hz}}}^{{\rm{M}}\to {\rm{Cs}}}$/*I*^2^ on the metal ion species, as shown in Fig. 6, is different from that of ${{S}}_{{\rm{10Hz}}}^{{\rm{M}}}$/*I*^2^ in Fig. 4. The value of ${{S}}_{{\rm{10Hz}}}^{{\rm{M}}\to {\rm{Cs}}}$/*I*^2^ was replaced with the value of ${{S}}_{{\rm{10Hz}}}^{{\rm{Cs}}}$/*I*^2^ when the nanopore was fabricated in an alkali metal ionic aqueous solution. On the other hand, the value of ${{S}}_{{\rm{10Hz}}}^{{\rm{M}}\to {\rm{Cs}}}$/*I*^2^ remained the same as the value of ${{S}}_{{\rm{10Hz}}}^{{\rm{M}}}$/*I*^2^ when the nanopore was fabricated in an alkaline earth metal ionic aqueous solution. This result suggests that Mg and Ca ions were still adsorbed onto the silanol groups even after replacement with the alkali metal ionic aqueous solution. The method utilizing dielectric breakdown in alkaline earth metal chloride aqueous solutions is effective for reducing the low-frequency noise of the CsCl ionic current.

**Figure 7:**
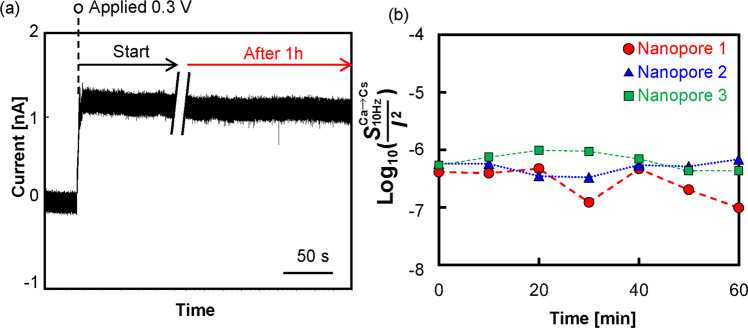
(**a**) Time trace of the CsCl ionic current flowing through the nanopore fabricated by utilizing dielectric breakdown in a CaCl_2_ aqueous solution. The current was measured at 0.3 V for 1 hour. (**b**) Time dependency of the low-frequency noise coefficient (${S}{}_{{\rm{10Hz}}}^{{\rm{Ca}}\to {\rm{Cs}}}$/*I*^2^) of the CsCl ionic current through the nanopore fabricated by utilizing dielectric breakdown in a CaCl_2_ aqueous solution. The data were acquired at 0.3 V in three different nanopores.

The long-term stability of the CsCl ionic current through a nanopore created via dielectric breakdown in a CaCl_2_ aqueous solution was also investigated (Fig. 7(a)). Apparently, the current remained stable at 1 hour after the start of the measurement. Figure 7(b) shows the time dependency of ${{S}}_{{\rm{10Hz}}}^{{\rm{Ca}}\to {\rm{Cs}}}$/*I*^2^ measured in three different nanopores. PSD analysis was performed every 10 min for 1 hour. For all three nanopores, the low-frequency noise magnitudes were lower than 10^−6^ at any point. There was no increase in the low-frequency noise magnitude even after the 1-hour measurement, and this result suggests that the alkaline earth metal ions were hardly desorbed from the silanol groups in the long-term measurement.

## Conclusion

In summary, we found that the magnitude of the low-frequency noise in the ionic current through a nanopore changes depending on the metal ion species in the aqueous solution. The low-frequency noise magnitude (${{S}}_{{\rm{10Hz}}}^{{\rm{M}}}$/*I*^2^) was attributed to the adsorption affinity of the metal ion for silanol groups. ${{S}}_{{\rm{10Hz}}}^{{\rm{M}}}$/*I*^2^ of the alkali metal ionic current was higher when ions had a higher adsorption affinity (in other words, ions with larger atomic numbers). This is likely attributed to the enhancement in the exchange reaction between alkali metal ions and protons on the silanol surface of the nanopore. The ${{S}}_{{\rm{10Hz}}}^{{\rm{M}}}$/*I*^2^ values of the alkaline earth metal ionic currents were lower than those of most alkali metal ionic currents. This likely occurs because the adsorption affinities of alkaline earth metal ions are high enough to be hardly desorbed from the silanol groups once they have been adsorbed, leading to a reduction in the exchange reaction between alkali earth metal ions and protons. In addition, even after replacing the alkaline earth metal chloride (MCl_2_) aqueous solution with the CsCl aqueous solution, the low-frequency noise magnitude (${{S}}_{{\rm{10Hz}}}^{{\rm{M}}\to {\rm{Cs}}}$/*I*^2^) remained low; that is, ${{S}}_{{\rm{10Hz}}}^{{\rm{M}}\to {\rm{Cs}}}$/*I*^2^ was almost equal to ${{S}}_{{\rm{10Hz}}}^{{\rm{M}}}$/*I*^2^ before the replacement. This indicated that alkaline earth metal ions continued being adsorbed onto the silanol surface even after aqueous solution replacement. Moreover, the value of ${{S}}_{{\rm{10Hz}}}^{{\rm{M}}\to {\rm{Cs}}}$/*I*^2^ remained low even 1 hour after the start of the measurement. This phenomenon can be useful for lowering the noise in nanopore measurements with CsCl aqueous solutions. For example, Goto *et al*.^25^ reported the identification of four types of homopolymers by blockade-current measurements using a nanopore in a CsCl aqueous solution. The identification accuracy could be improved if the nanopore was once immersed in a CaCl_2_ aqueous solution before the measurement.

The low-frequency noise magnitude is also thought to be related to the structure of the nanopore because the number of the exchange reaction sites changes depending on the area of the nanopore surface. For example, the low-frequency noise magnitude might change depending on the thickness of the nanopore while the diameter of the nanopore is set constant. The dependence of the low frequency noise on the thickness of the nanopore will be addressed in our future work.

We believe that the findings in this study regarding the dependence of low-frequency noise on the metal ion species are crucial in the development of high-precision nanopore sensors.

## Methods

### Fabrication of the nanopores

A nanopore was fabricated in a 6-nm-thick SiN_x_ membrane with a square area of approximately 600 × 600 nm^2^. The fabrication process of the membrane chip was the same as that described in our previous report^23^ except for the thickness of the SiN_x_ membrane. The SiN_x_ membrane was sandwiched between two reservoirs (each with a volume of 90 μL), including 1 mol/L MCl_x_ aqueous solutions (M = Li, Na, K, Rb, Cs, Mg, and Ca) with Tris-EDTA buffer (10 mM Tris-HCl and 1 mM EDTA) at a neutral pH of approximately 7.5. Two Ag/AgCl electrodes were immersed in both solutions. To fabricate a nanopore with a diameter of 1–2 nm via dielectric breakdown, multilevel pulse voltage injection (MPVI)^22^ was performed on the membrane with a 41501B SMU and pulse generator expander (Agilent Technologies, Inc., Santa Clara, CA) and a 4156B precision semiconductor parameter analyzer.

In the MPVI procedure, a high-voltage pulse (*V*_h_) is applied between the two electrodes to fabricate a nanopore via dielectric breakdown, and a low voltage (*V*_l_) is applied to verify whether the nanopore has been created. *V*_h_ and *V*_l_ were set to 6 and 0.1 V, respectively. The duration of the n_th_-high-voltage pulse was set as:

$${{\rm{t}}}_{{\rm{n}}}={10}^{-3+\frac{{\rm{n}}-1}{12}}-{10}^{-3+\frac{{\rm{n}}-2}{12}}\,{\rm{for}}\,{\rm{n}}\ge 2$$

$${{\rm{t}}}_{1}={10}^{-3}\,{\rm{for}}\,{\rm{n}}=1$$

The accumulated time (*t*_sum_ = Σ*t*_n_) of the applied pulse durations for nanopore creation was less than 1 s.

### Measurement of the ionic current

Measurement of the ionic current flowing through the nanopore was started within 1 min after nanopore fabrication. The ionic current measurements were performed using a patch-clamp amplifier (Axopatch 200B, Axon Instruments, Union City, CA). The detected current was low-pass filtered with a cut-off frequency of 10 kHz using a four-pole Bessel filter and then digitized with an NI USB-6281 18-bit DAQ AD converter (National Instruments, Austin, TX) at a sampling rate of 50 kHz. Finally, the current was recorded on the hard disk of a personal computer. All the measurements described above were performed at room temperature. The PSDs were obtained by performing 65536-point fast Fourier transformation (FFT) and averaging the PSDs more than 10 times.

## Supplementary information

Supplementary information.

## Data Availability

All data generated during this study are included in this published article and its supplementary information files. The datasets analysed during the study are available from the corresponding author on reasonable request.
