# Supplementary material for: Low-frequency noise induced by cation exchange fluctuation on the wall of silicon nitride nanopore
Source: Sci Rep. 2020 May 26;10:8662. doi: 10.1038/s41598-020-65530-y (PMC7250840; doi:10.1038/s41598-020-65530-y)
Supplement: Supplementary file 1 — Supplementary information. [file 41598_2020_65530_MOESM1_ESM.pdf]

Supplementary Information

Low-frequency noise induced by cation exchange fluctuation on the wall of silicon nitride nanopore

Kazuma Matsui[1,2], Yusuke Goto[1,2], Itaru Yanagi[1], Rena Akahori[1], Michiru Fujioka[3], Takeshi Ishida[1], Takahide Yokoi[1], Tatsuo Nakagawa[1], and Ken-ichi Takeda[1]

[1]Center for Technology Innovation – Healthcare, Research and Development Group, Hitachi, Ltd., 1-280 Higashi-Koigakubo, Kokubunji, Tokyo 185-8601, Japan

[2]Department of Biotechnology and Life Science, Tokyo University of Agriculture and Technology, Koganei, Tokyo, 184-8588, Japan

[3]Bio Systems Design Department, Hitachi High-Tech Corporation, 882 Ichige, Hitachinaka, Ibaraki, 312-8504, Japan

SI-A: Bias polarity dependence in low-frequency noise

As shown in Fig. S1(a), we examined the ionic current through the nanopore when *cis* chamber and *trans* chambers were filled with CsCl and LiCl aqueous solution, respectively. The ionic current was measured when 0.2 V was applied between *cis* and *trans* electrodes. The voltage in the *cis* chamber was set at 0 V, and the voltage in the *trans* chamber was set at either (i) a positive (0.2 V) or (ii) a negative (-0.2 V) voltage. Fig. S1(b) shows power spectral densities of the ionic currents flowing through the nanopore when the positive (blue) and negative (red) bias were set to the *trans* electrode. In the case of (i) where the positive bias was applied to the *trans* electrode, *i.e.*, Li ionic current flowed through the nanopore, the low-frequency noise was relatively low. On the other hand, in the case of (ii) where the negative bias was applied, *i.e.*, Cs ionic current flowed through the nanopore, the low-frequency noise was much higher than in the case of (i). This result also supports our hypothesis wherein the magnitude of low-frequency noise depends on the cationic species in the nanopore.

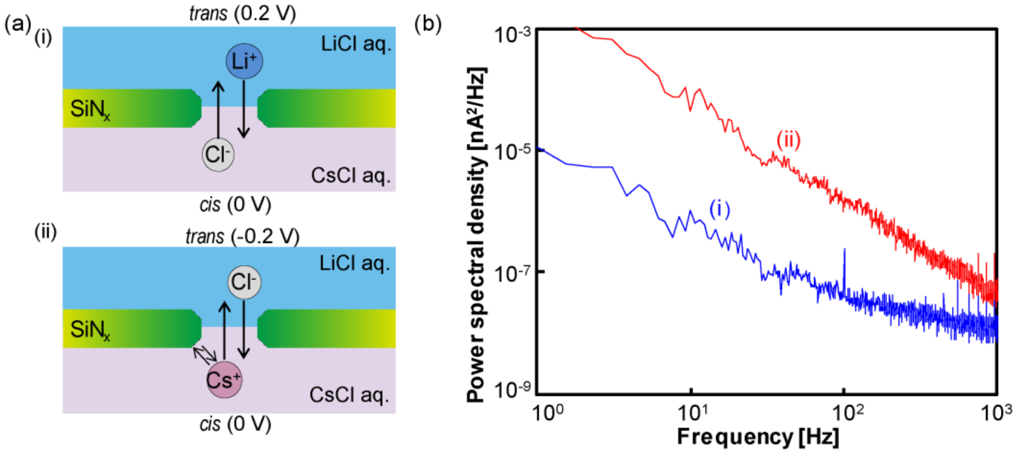

Fig. S1. (a) Schematic images of the ionic-current measurement when one side of the chambers (*trans*) was filled with LiCl aqueous solution while the other side (*cis*) was filled with CsCl aqueous solution. A voltage of the *cis* electrode was set at 0 V. An ionic current was measured when (i) a positive bias (0.2 V) and (ii) a negative bias (-0.2 V) were set to the *trans* electrode. (b) PSDs of the ionic currents through the nanopore when the *trans* voltage was 0.2 V (blue) and -0.2 V (red).

SI-B: Time dependency of the low-frequency noise

As shown in Fig. 5, we examined the time dependency of the low-frequency noise magnitude. Each nanopore was fabricated via dielectric breakdown in 1 mol/L CsCl and LiCl aqueous solutions. Figs. S2 and S3 show the PSDs and time traces of the currents over 240 s measured in CsCl and LiCl aqueous solutions. While the LiCl ionic current remained stable at any point, the CsCl ionic current largely fluctuated. The PSDs of Cs were higher than those of Li.

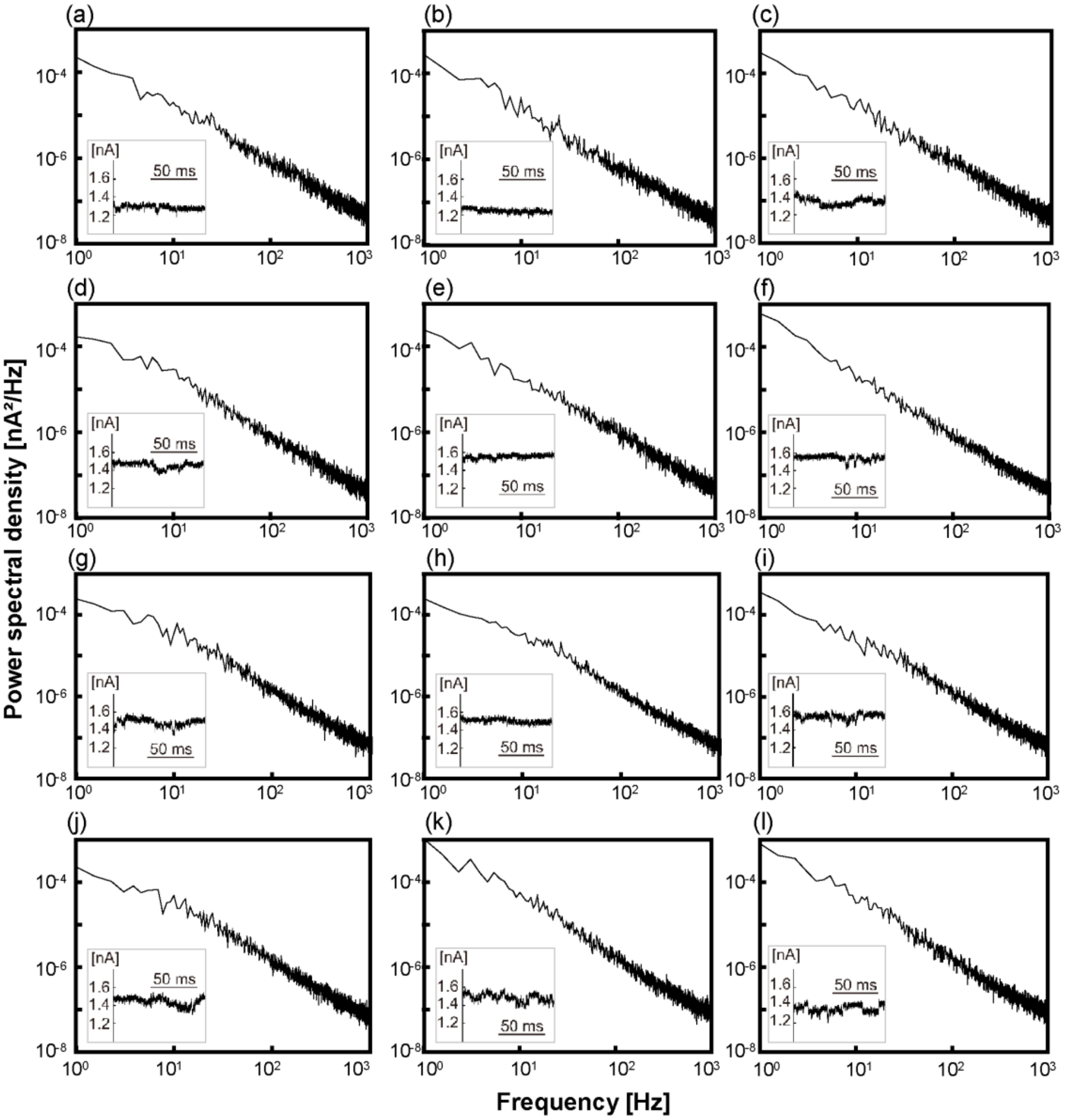

Fig. S2. PSDs of the ionic current flowing through the nanopore in the CsCl aqueous solutions at 0.2 V. Each PSD analysis in (a-l) was performed at 20-s intervals. The voltage applied was 0.2 V. The insets show the time traces of the ionic currents at (a) 0 s, (b) 20 s, (c) 40 s, (d) 60 s, (e) 80 s, (f) 100 s, (g) 120 s, (h) 140 s, (i) 160 s, (j) 180 s, (k) 200 s, and (l) 220 s after the start of the measurement.

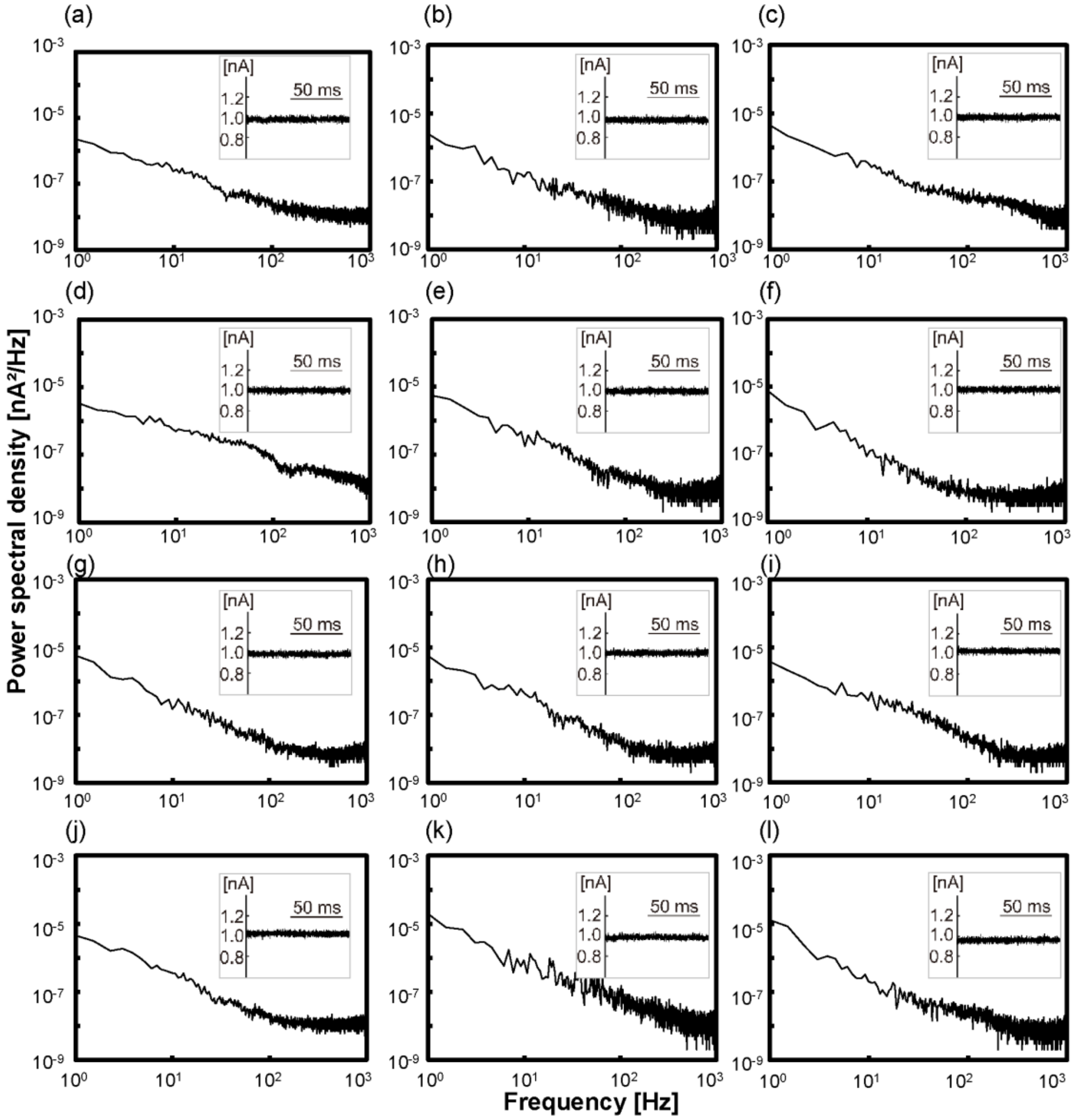

Fig. S3. PSDs of the ionic current flowing through the nanopore in the LiCl aqueous solutions at 0.2 V. Each PSD analysis in (a-l) was performed at 20-s intervals. The voltage applied was 0.2 V. The insets show the time traces of the ionic currents at (a) 0 s, (b) 20 s, (c) 40 s, (d) 60 s, (e) 80 s, (f) 100 s, (g) 120 s, (h) 140 s, (i) 160 s, (j) 180 s, (k) 200 s, and (l) 220 s after the start of the measurement.
